# Considerations for the practical management of cardiovascular risk with Bruton’s tyrosine kinase inhibitors for patients with chronic lymphocytic leukemia

**DOI:** 10.1093/oncolo/oyaf237

**Published:** 2025-07-30

**Authors:** Daniel Lenihan, Michelle Bloom, Robert Copeland-Halperin, Matthew R Fleming, Michael Fradley, Rupal O’Quinn, Seema A Bhat

**Affiliations:** Cardio-Oncology Center of Excellence, Cardiac Rehabilitation, and Heart Failure Clinic, Saint Francis Healthcare System, MO 63703, United States; Cardio-oncology program, NYU Langone Health, New York, NY 11501, United States; Cardiology, Northwell Health, New York, NY 11040, United States; Cardiovascular Medicine, Vanderbilt University Medical Center, Nashville, TN 37232, United States; Cardio-oncology, Hospital of the University of Pennsylvania, Philadelphia, PA 19104, United States; Cardiovascular Division, Perelman School of Medicine, University of Pennsylvania, Philadelphia, PA 43210, United States; Hematology, The Ohio State University Comprehensive Cancer Center, Columbus, OH 43210, United States

**Keywords:** chronic lymphocytic leukemia, Bruton’s tyrosine kinase inhibitor, cardiovascular, arrhythmia, hypertension, hemorrhage

## Abstract

**Background:**

Bruton’s tyrosine kinase inhibitors (BTKis) are central to the medical management of chronic lymphocytic leukemia. However, accumulating data suggest an important association with cardiovascular (CV) adverse events (AEs), including arrhythmias, hypertension, and bleeding, in patients with chronic lymphocytic leukemia and other hematological malignancies treated with this therapeutic class. Data from comparative trials with BTKis suggest second-generation agents, for example, acalabrutinib and zanubrutinib, may be associated with fewer CV AEs than first-in-class BTKi ibrutinib.

**Methods:**

PubMed and the proceedings of key hematology congresses were searched for relevant information using broad search terms, including chronic lymphocytic leukemia, BTKi, and toxicity.

**Results:**

When managing patients with chronic lymphocytic leukemia, screening before and during treatment to assess CV risk is suggested to guide decision-making. Due to the increased toxicity with ibrutinib, the second-generation BTKis are now preferred (per the NCCN Clinical Practice Guidelines in Oncology [NCCN Guidelines]). For patients with a high CV risk, the decision between second-generation BTKi or a time-limited alternative, like venetoclax plus an anti-CD20 monoclonal antibody, should be made on an individual basis after patient consultation and consideration of the presenting characteristics of chronic lymphocytic leukemia in any given patient. The management of anticoagulant/antiplatelet medication during BTKi treatment requires specific attention, with coexistent medications being carefully assessed before starting a BTKi to reduce the risk of bleeding. For patients with a new-onset or worsening CV events during BTKi therapy, management may involve temporarily stopping the BTKi or switching to another class of therapy. To ensure the best outcomes, a collaborative care approach is essential, and some patients may need to be referred to a cardiologist/cardio-oncologist for specialist management.

**Conclusion:**

Baseline and ongoing CV risk assessment, careful monitoring, management, and a multidisciplinary team approach are all critical to ensure optimal oncologic and CV outcomes for patients with chronic lymphocytic leukemia receiving BTKis.

Implications for Practice BTKis are a mainstay of treatment for patients with chronic lymphocytic leukemia, but data suggest an increased risk of CV AEs with these therapies. This is a particular concern because of the older average age of patients with chronic lymphocytic leukemia and their potential CV comorbidities. It is essential that healthcare professionals understand the relative risk of CV AEs with the different types of BTKis and the importance of initial screening and on-treatment monitoring. Increasing awareness of these factors can improve treatment decision-making for chronic lymphocytic leukemia and optimize patient outcomes.

## Introduction

The introduction of Bruton’s tyrosine kinase inhibitors (BTKis) has revolutionized the treatment of patients with chronic lymphocytic leukemia chronic lymphocytic leukemia, offering improved efficacy and tolerability over chemoimmunotherapeutic regimens. Ibrutinib, an irreversible (covalent) BTKi, was the first of this class to be approved. Subsequently, second-generation, irreversible BTKis, acalabrutinib and zanubrutinib, were approved by the Food and Drug Administration (FDA) for the management of chronic lymphocytic leukemia. Pirtobrutinib and nemtabrutinib, reversible (noncovalent) BTKis, are under investigation for chronic lymphocytic leukemia^1^^,^^2^; pirtobrutinib was granted accelerated approval by the FDA for patients with CLL/small lymphocytic lymphoma who have received at least 2 prior lines of therapy, including a BTKi and a B-cell lymphoma (BCL)-2 inhibitor. Approved BTKis, given as continuous therapy for a prolonged time, and fixed-duration venetoclax (BCL-2 inhibitor) in combination with an anti-CD20 monoclonal antibody (obinutuzumab or rituximab) are the mainstay treatments for front-line and relapsed/refractory chronic lymphocytic leukemia.^3^^,^^4^ National Comprehensive Cancer Network Clinical Practice Guidelines in Oncology [NCCN Guidelines] include acalabrutinib, alone or in combination with obinutuzumab, or zanubrutinib, as preferred first-line BTKi regimens, and acalabrutinib in combination with venetoclax with or without obinutuzumab as a fixed-duration regimen was recently added as a preferred first-line treatment.^5^

BTKis exert their effects by directly targeting and blocking the activity of BTK, an enzyme crucial for B-cell receptor signaling, thereby preventing the proliferation and survival of B cells by disrupting their activation pathway, which is critical for the development of B-cell malignancies and autoimmune diseases.^6^ BTKis can bind to other kinases causing off-target effects that may manifest as a variety of adverse events (AEs). The degree of off-target binding may influence the type, frequency, and severity of AEs; acalabrutinib and zanubrutinib may be more specific for BTK than ibrutinib with fewer off-target effects, resulting in a decreased incidence of AEs.^7^ Accumulating data indicate an increased risk of cardiovascular (CV) AEs, including arrhythmias (particularly atrial fibrillation/flutter, hypertension, and bleeding) secondary to BTKis. The risk of CV AEs is a concern, particularly because of the older average age of patients with chronic lymphocytic leukemia (approximately 70 years).

Here, we discuss the relative risk of CV AEs with various BTKis and provide practical suggestions for initial screening for underlying CV risks, treatment choice based on CV risk factors and pre-existing CV disease, CV monitoring during chronic lymphocytic leukemia therapy, and the optimal management of patients with CV AEs.

## Materials and methods

In the last quarter of 2023, a meeting of 11 US-based cardio-­oncologists and oncologists was convened to discuss the clinical implications and management of BTKi-induced CV AEs. At the meeting, a need was identified for practical recommendations for the chronic lymphocytic leukemia-treating community to mitigate the risk of and to manage CV AEs. Eight of the meeting attendees subsequently developed this article. PubMed and the proceedings of key hematology congresses were searched for relevant information using broad search terms including chronic lymphocytic leukemia, BTKi, and toxicity.

### Risk of CV AEs with different BTKis

Assessing the risk of CV AEs with different BTKis from key chronic lymphocytic leukemia clinical trials is challenging as outcomes may be affected by various factors. CV risk at baseline is not typically defined and inclusion criteria relevant to CV risk can differ among trials. In the ELEVATE-RR (acalabrutinib vs ibrutinib)^8^^,^^9^ and ALPINE (zanubrutinib vs ibrutinib)^10^ trials, significant CV disease was an exclusion criterion and was specified in both trials as myocardial infarction within 6 months, a history of uncontrolled, symptomatic, or clinically significant arrhythmias (eg, sustained ventricular tachycardia, ventricular fibrillation, Torsades de Pointes), New York Heart Association class III or IV heart failure, or QT corrected (QTc) >480 milliseconds (corrected for heart rate in ALPINE: QTcF); the ALPINE trial also excluded uncontrolled hypertension, unstable angina within 3 months, and a history of Mobitz II second-/third-­degree heart block without a permanent pacemaker.^10^ Patient characteristics at baseline are also a consideration, particularly median age and the number of prior therapies. Data from a pooled analysis of patients who received acalabrutinib monotherapy showed that acalabrutinib-associated atrial fibrillation/flutter was more frequent with increasing age (age <65 years, 2% [*n *=* *7/288]; 65 to <75 years, 6% [*n *=* *19/318]; ≥75 years, 8% [*n *=* *12/156]).^11^ In addition, the number of prior therapies was significantly associated with new or worsening hypertension on acalabrutinib in a multivariate analysis of patients from a large US-based Comprehensive Cancer Center treated with acalabrutinib from 2014 to 2020 for any lymphoid malignancy.^12^ One possible concern is the lack of diversity in specific clinical trials, particularly sex, ethnicity, and socioeconomic status, perhaps not reflecting a broad population of patients. Because data with acalabrutinib, zanubrutinib, and ibrutinib show an increasing cumulative incidence of CV AEs over time,^8^^,^^10^ the duration of treatment and follow-up is important. Finally, the Common Terminology Criteria for Adverse Events (CTCAE) criteria routinely used in oncology trials are limited in the detailed description of CV AEs (Table 1).^13^ Because of these limitations, the relative impact of BTKis on CV AEs is best evaluated using data from the few clinical trials in which BTKis have been compared directly (Table 2)^8^^,^^10^ or from large retrospective datasets and meta-analyses.

**Table 1.** Overview of National Cancer Institute Common Terminology Criteria for atrial fibrillation/flutter and hypertension.^13^

CTCAE term	Grade 1	Grade 2	Grade 3	Grade 4	Grade 5
**Atrial fibrillation** **Atrial flutter**	Asymptomatic, intervention not indicated	Non-urgent medical intervention indicated	Symptomatic, urgent intervention indicated; device (eg, pacemaker); ablation; new onset AF	Life-threatening consequences; embolus requiring urgent intervention	Death
**Hypertension (adult)**	Systolic blood pressure 120-139 mm Hg or diastolic blood pressure 80-89 mm Hg	Systolic blood pressure 140-159 mm Hg or diastolic blood pressure 90-99 mm Hg if previously within normal limits; change in baseline medical intervention indicated; recurrent or persistent (≥24 h); symptomatic increase by >20 mm Hg (diastolic) or to >140/90 mm Hg; monotherapy indicated initiated	Systolic blood pressure ≥160 mm Hg or diastolic blood pressure ≥100 mm Hg; medical intervention indicated; more than one drug or more intensive therapy than previously used indicated	Life-threatening ­consequences (eg, malignant hypertension, transient or permanent neurologic deficit, hypertensive crisis); urgent intervention indicated	Death
Hemorrhage^a^ (eg, bronchopulmonary, gastric, upper/lower gastrointestinal, hepatic, and pancreatic)	Mild symptoms; intervention not indicated	Moderate symptoms; invasive intervention not indicated	Transfusion indicated; invasive intervention indicated; hospitalization	Life-threatening consequences; intubation or urgent intervention indicated	Death

a Please refer to the reference for information about hemorrhage in specific sites.^13^

**Table 2.** Summary of CV-related toxicity data from the ELEVATE-RR and ALPINE head-to-head BTKi clinical trials in patients with chronic lymphocytic leukemia.^8^^,^^10^

	**ELEVATE-RR^8^Relapsed/refractory chronic lymphocytic leukemia, ≥18 years with del(17)(p13.1) and/or del(11)(q22.3)Median follow-up 40.9 months**		**ALPINE^10^Relapsed/refractory chronic lymphocytic leukemia/small lymphocytic leukemia, ≥18 yearsMedian follow-up 29.6 months**	
	Acalabrutinib (*n *=* *266)	Ibrutinib (*n *=* *263)	Zanubrutinib (*n *=* *324)	Ibrutinib (*n *=* *324)
**Atrial fibrillation/flutter, any grade, %**	9	16	5	13
**Atrial fibrillation/flutter, grade ≥3, %**	5	4	2	4
**Hypertension, any grade, %**	9	23	23	23
**Hypertension, grade ≥3, %**	4	9	15	14
**Hemorrhage/bleed, any grade, %**	38	51	42	41
**Hemorrhage/bleed, grade ≥3, %**	4	5	3	4
**Major hemorrhage, any grade, %**	5^a^	5^a^	4^b^	4^b^
**Major hemorrhage, grade ≥3, %**	4^a^	5^a^	3^b^	4^b^

a Defined in ELEVATE-RR as any hemorrhagic event that was serious, grade ≥3, or a CNS hemorrhage (any grade).

b Defined in ALPINE as subdural hematoma (preferred term), subdural hemorrhage (preferred term), all hemorrhage (preferred term) if AE system organ class is “Nervous system disorders,” or serious or grade ≥3 hemorrhage (preferred term) if AE system organ class is not “Nervous system disorders.”

Abbreviations: AE, adverse event; BTKi, Bruton tyrosine kinase inhibitor; CNS, central nervous system; CV, cardiovascular.

#### Atrial fibrillation

Outcomes from major trials and retrospective analyses show an association between the use of ibrutinib and the incidence of atrial fibrillation/flutter.^8^^,^^10^^,^^11^^,^^14–17^ In a retrospective analysis of 4958 patients with chronic lymphocytic leukemia, 6% of whom received ibrutinib, a 3.65-fold increased risk of atrial fibrillation/flutter was observed with ibrutinib.^14^ Similarly, retrospective analysis of CV AEs in 515 patients with chronic lymphocytic leukemia treated with either first-line ibrutinib or other non-ibrutinib therapy showed ibrutinib was highly associated with CV AEs, regardless of baseline CV risk.^15^ The odds ratios of atrial fibrillation/flutter with ibrutinib were 3.02 and 2.46 compared with other non-ibrutinib therapy, respectively.^15^

Data from clinical trials, meta-analyses, and retrospective studies with the second-generation BTKis acalabrutinib or zanubrutinib show that they are also associated with CV AEs but often at a lower frequency than with ibrutinib.^8^^,^^10^^,^^11^^,^^16^^,^^17^ In the ELEVATE-RR and ALPINE comparative BTKi trials, data show a lower incidence of any-grade atrial fibrillation/flutter with the second-generation BTKis than ibrutinib, but there was little or no difference in grade ≥3 atrial fibrillation/flutter with acalabrutinib versus ibrutinib in ELEVATE-RR (Table 2).^8^^,^^10^ In ALPINE, the rate of grade 3 atrial fibrillation/flutter with ibrutinib was higher than with zanubrutinib, although the difference was less pronounced than that in the ASPEN trial, a phase 3 study in patients with a different B-cell malignancy (Waldenström macroglobulinemia) (any-grade, 23.5% vs 7.9%; grade ≥3, 8.2% vs 2.0%, respectively).^17^ In a pooled analysis of 10 studies with zanubrutinib in patients (*N *=* *1550) with B-cell malignancies and a pooled analysis of head-to-head trials comparing zanubrutinib with ibrutinib (ASPEN and ALPINE), the overall incidence of atrial fibrillation/flutter was lower with zanubrutinib than ibrutinib. In ALPINE and ASPEN, despite a similar prevalence of preexisting CV events, atrial fibrillation/flutter incidence rates (6.1% vs 15.6%) and exposure-adjusted incidence rates (0.2 vs 0.64 persons per 100 person-months) were lower with zanubrutinib than with ibrutinib.^18^

Findings from a meta-analysis of 833 patients treated with acalabrutinib or control treatment in randomized clinical trials showed a considerable trend toward a statistically significant increased risk of any-grade atrial fibrillation/flutter with acalabrutinib (risk ratio, 2.65; *P* = .05).^16^ In a pooled analysis of 762 patients who received acalabrutinib, there was a 5% incidence of atrial fibrillation/flutter, with 18% of these patients having CV risk factors before treatment.^11^

#### Other arrhythmias and sudden cardiac death

Although not initially recognized, emerging evidence suggests that BTKis may be associated with an increased risk of serious ventricular arrhythmias and sudden cardiac death.^19–23^ In the GLOW trial of fixed-duration ibrutinib + venetoclax versus chlorambucil + obinutuzumab, there were 4 sudden cardiac deaths during treatment on the ibrutinib + ventoclax arm and all were patients with a Cochin Risk Index Score score of ≥10 or an Eastern Cooperative Oncology Group performance status score of 2. Of note, all 4 patients had a history of hypertension, CV disease, and/or diabetes. There were no sudden cardiac deaths in the control arm.^22^ In the ALLIANCE trial of ibrutinib, either alone or in combination with rituximab, versus bendamustine + rituximab, there were 5 sudden deaths on the ibrutinib arm, 2 on the ibrutinib + rituximab arm, and 2 on the bendamustine + rituximab arm.^23^ In 2 retrospective analyses, the incidence of ventricular arrhythmias and sudden cardiac deaths among ibrutinib-treated patients was greater than that in similar, non–ibrutinib-treated patients.^19^^,^^20^ More recently, retrospective data indicated a greater number of incident ventricular arrhythmias (comprising symptomatic premature ventricular tachycardia, sustained ventricular tachycardia, and ventricular fibrillation) and sudden cardiac deaths among acalabrutinib-treated patients than in similar, non–BTKi-treated patients (394 per 100 000 person years vs 48.1 per 100 000 person-years).^21^ In contrast, an analysis of 5 clinical trials (>1200 patients) indicated a low risk of ventricular arrythmias and sudden cardiac deaths with acalabrutinib,^24^ and results from a pooled analysis showed no sudden cardiac deaths among 762 acalabrutinib-treated patients.^11^

In head-to-head trials, non-atrial fibrillation/flutter arrhythmias were rare, making it difficult to conclude a class effect for any arrythmias beyond atrial fibrillation/flutter. In ELEVATE-RR, 3 ibrutinib-treated patients had a ventricular arrythmia, and there was 1 sudden cardiac death.^8^ In ALPINE, non-atrial fibrillation/flutter arrhythmias occurred in <1% of patients (any grade) in either arm, except sinus bradycardia (zanubrutinib, 1.5%; ibrutinib, 2.2%) and sinus tachycardia (zanubrutinib, 1.2%; ibrutinib, 0.3%).^10^ Six patients in the ibrutinib arm died due to cardiac AEs (cardiac arrest, *n *=* *2; myocardial infarction, *n *=* *2; and heart failure, *n *=* *2).^10^ Findings from a pooled analysis of ALPINE and ASPEN showed that the incidence of symptomatic ventricular arrhythmias was low for zanubrutinib (0.7%) and ibrutinib (1.7%), with a numerically lower exposure-adjusted incidence rates (0.02 vs 0.06 persons per 100 person-months, respectively) for zanubrutinib.^18^

#### Hypertension

The relationship between BTKis and hypertension is important since hypertension in the general population is likely the most important risk factor for incident atrial fibrillation/flutter.^25^ Additionally, hypertension can potentially exacerbate the risk of bleeding and stroke.^26^ Data show an association between BTKis and hypertension, with some studies indicating that the incidence of hypertension with second-generation BTKis, especially acalabrutinib, may be lower than with ibrutinib.^8^^,^^17^ In a retrospective analysis, the odds ratios of any hypertension with ibrutinib vs. other non-ibrutinib therapy were 3.66 and 2.13, respectively (*N *=* *515).^15^ Outcomes from a meta-analysis of 8 randomized trials (*N *=* *2580) showed ibrutinib was associated with a significant increase in hypertension (risk ratio, 2.82; *P* < .001).^27^ Real-world data from a large US-based Comprehensive Cancer Center cohort of 280 consecutive patients with lymphoid malignancies initiated on acalabrutinib showed that 59.2% of patients developed new or worsening hypertension after a median follow-up of 41 months.^12^ In contrast, data from a meta-analysis of 833 acalabrutinib-treated patients showed no significant increase in the risk of hypertension.^16^ Outcomes among the comparative clinical trials differed: data from the ELEVATE-RR^8^ trial showed a greater incidence of hypertension with ibrutinib than acalabrutinib, but in the ALPINE trial, the incidence was similar with ibrutinib and zanubrutinib^10^ (Table 2). In ASPEN, the comparative trial in patients with Waldenström macroglobulinemia, the incidence of hypertension was higher with ibrutinib than zanubrutinib (any grade, 25.5% vs 14.9%; grade ≥3, 20.4% vs 9.9%).^17^ In a pooled analysis of the ASPEN and ALPINE trials, the rate of hypertension was 21.9% with zanubrutinib versus 23.5% with ibrutinib.^18^

#### Bleeding

Results of several analyses suggest an increased risk of any-grade and major bleeding with BTKis (Table 2).^8^^,^^10^ A retrospective analysis of 4958 patients with chronic lymphocytic leukemia, 6% of whom received ibrutinib, showed a 4.92-fold increased risk of bleeding and a 7.49-fold increased risk of major bleeding with ibrutinib.^14^ In a retrospective analysis of 289 patients treated with acalabrutinib for a long duration (median exposure was 40.8 months, range 0-81.6 months), the incidence of bleeding was higher than expected (83%) based on published literature, although most bleeds were not considered clinically relevant (59%), and the incidence of major bleeds was 6%.^28^ In the ELEVATE-RR trial, there was a significantly higher incidence of bleeding events (which included contusion and epistaxis) with ibrutinib vs. acalabrutinib, while the incidence of major bleeding (defined as any hemorrhagic event that was serious, grade ≥3 in severity, or that was a central nervous system [CNS] hemorrhage of any severity) was similar.^8^ In the ALPINE trial, bleeding events, including major bleeding, occurred with similar frequency among patients receiving zanubrutinib and ibrutinib.^10^

Bleeding risk with BTKis is of particular importance in patients with preexisting or new onset atrial fibrillation/flutter requiring anticoagulation or antiplatelet therapy, which can further increase bleeding risk. Data support the increased risk of BTKi-associated bleeding with anticoagulation or antiplatelet therapy. This creates a challenge for many patients with chronic lymphocytic leukemia and atrial fibrillation/flutter. An international registry study was designed to evaluate the outcomes of 202 patients with different types of cancer who were receiving concurrent direct oral anticoagulants and targeted anticancer therapies.^29^ The findings showed that the cumulative incidence of major bleeding events was higher in patients receiving BTKis (10%) than in patients receiving alternative drugs/drug classes, including vascular endothelial growth factor receptor inhibitors, palbociclib, and epidermal growth factor receptor/anaplastic lymphoma kinase inhibitors.^29^ In a pooled analysis of 762 patients with chronic lymphocytic leukemia receiving acalabrutinib, among 299 patients receiving on-study anticoagulant treatment for any reason, 12 (4%) had a major hemorrhage event.^11^ Another study of ibrutinib-treated patients showed that among those with a bleeding event, 30.2% had received anticoagulation in the preceding year, while the rate of anticoagulant use in patients without bleeding was 15.8%.^14^ The mechanism of BTKi-associated bleeding risk is not fully understood, but is believed to involve both on- and off-target kinase inhibition, particularly as BTK mediates platelet aggregation through effects on collagen receptor glycoprotein VI.^30^^,^^31^

The potential for stroke in patients receiving BTKis is of concern because of the increased risk of bleeding with BTKis, often-extensive anticoagulant use, and known association between stroke and atrial fibrillation/flutter. Few embolic stroke events have been reported in clinical trials, although this has been infrequently evaluated in retrospective analyses. When considering the risks and benefits of different treatments, particularly with relation to anticoagulation for embolic stroke prevention, it is important to distinguish major bleeding, particularly CNS hemorrhage, from minor, grade 1/2 bleeding. Although minor bleeding might have a negative impact on patients and affect their quality of life, it rarely leads to discontinuation of therapy. Thus, although the incidence of major bleeding is relatively low, the approximately 40%-50% incidence of any-grade bleeding events^8^^,^^10^ can be relevant for some patients. Unnecessary antiplatelet or anticoagulant therapy should be discontinued to minimize such a quandary. Finally, a better understanding of bleeding and bleeding risk among BTKi-treated patients can be improved if future clinical trials include detailed information about the detection and classification of minor and major bleeding and whether these events led to therapy discontinuation. Furthermore, determining the associated risk of stroke in these patients versus the general population, and whether prophylaxis is needed, is important.

#### Reversible, noncovalent third-generation BTKis

Pirtobrutinib and nemtabrutinib are noncovalent, reversible BTKis and have been evaluated in patients with relapsed/refractory chronic lymphocytic leukemia.^1^^,^^2^ In the phase 1/2 BRUIN trial, among 317 patients treated with pirtobrutinib, treatment-emergent any-grade and grade ≥3 atrial fibrillation/flutter occurred in 2.8% and 1.2% of the 733-patient safety population, respectively; any-grade hypertension occurred in 9.2% of patients (2.3% grade ≥3); any-grade bleeding in 36.0% (2.2% grade ≥3); and any-grade hemorrhage in 19.1% (2.2% grade ≥3).^1^ No drug-related ventricular fibrillation, ventricular tachycardia, or sudden cardiac death were reported.^1^ In the BELLWAVE-001 phase 1/2 trial, among 47 patients with relapsed/refractory hematologic malignancies treated with nemtabrutinib, 1 patient had grade 3 AF, 3 had grade 1/2 bleeding, and 32% of patients had grade 2/3 hypertension.^2^ Early-phase data with pirtobrutinib suggest an encouraging CV safety profile, potentially due to the high BTK selectivity and limited off-target inhibition.^1^ Data from larger, ideally comparative trials, including the ongoing BRUIN CLL-314 phase 3 trial of pirtobrutinib versus ibrutinib,^32^ are needed to determine the CV profile of these agents relative to other BTKis.

In summary, for most patients, the relative CV risk is an important consideration when selecting therapy, and data suggest that the second-generation BTKis are preferable over ibrutinib. This is reflected in the updated NCCN Guidelines in 2022 when the panel consensus resulted in a change from listing ibrutinib under “preferred regimens” to “other recommended regimens.”^5^ However, there are no directly comparative data on which to base a judgment of superiority of acalabrutinib over zanubrutinib or vice versa. In future trials, CV AEs should be carefully defined to allow clinicians to make informed choices between these therapies.

#### Risk factors for CV AEs with BTKis

The prevalence of atrial fibrillation/flutter is higher in patients with chronic lymphocytic leukemia than in the general population.^33–35^ The lifetime risk of atrial fibrillation/flutter in the general population is reported to range from 17% to 26% for men and from 21% to 23% for women aged 40 years or older.^33^ In the United States, the prevalence of atrial fibrillation/flutter in people older than 65 years is estimated to be 9%.^34^ Among 2292 patients without a history of atrial fibrillation/flutter at chronic lymphocytic leukemia diagnosis, 6.1% developed incident atrial fibrillation/flutter during follow-up for their chronic lymphocytic leukemia (median follow-up, 7.3 years), with an incidence of approximately 1% per year.^35^

Data also suggest that patients with cancer and pre-existing CV disease (eg, hypertension, atrial fibrillation/flutter, or prior myocardial infarction) are more likely to have a CV AE during treatment with either ibrutinib or a second-generation BTKi.^8^^,^^11^^,^^36^^,^^37^ Factors shown to increase the risk of CV AEs with BTKis include age,^38^ sex,^38^ diabetes,^38^ kidney disease,^38^ smoking status,^38^ CYP3A4 inhibitor use,^39^ baseline systolic blood pressure,^39^ and Black ancestry.^12^ Identifying the presence of these factors before initiation of chronic lymphocytic leukemia therapy is imperative for the assessment of CV risk. Ongoing risk stratification and monitoring throughout treatment are essential.

## Considerations for selecting first-line chronic lymphocytic leukemia therapy

CV screening before and throughout chronic lymphocytic leukemia therapy is imperative due to the clear relationship between BTKis and CV AEs. NCCN Guidelines recommend considering a CV risk assessment before starting a covalent BTKi.^5^ However, in clinical practice, this may not be a common practice and is an area for improvement. The importance of ongoing monitoring of patients on therapy cannot be overstated, both because of the association of higher CV AE risk with increasing duration of therapy, coupled with the aging of patients while on long-term treatment. In addition, AEs like hypertension, can take months to develop and may result in atrial fibrillation/flutter. Recommendations for assessments before and during BTKi therapy, adapted from the 2022 European Society of Cardiology Guidelines on Cardio-Oncology, can be performed by the oncology team and are provided in Table 3.^40^

**Table 3.** Recommendations for pretreatment and on-treatment screening for CV risk.^40^

Assessment	Baseline/pretreatment	On-treatment clinic visits	On-treatment at home
**Age, sex, and full medical history for prior CV disease**	✓		
**Family history of premature/early CVD**	✓		
**Review of any CV conditions**	✓	✓	
**Review of all CV medications**	✓	✓	
**Smoking status**	✓	✓	
**Presence of diabetes, lipid profile**	✓	✓ (Intermittent)	
**Blood pressure monitoring**	✓	✓	✓ Weekly^a^ For high-risk patients/those with a history of hypertension, consider daily assessment for first 3 weeks of BTKi treatment, and weekly thereafter
**Measurement of left ventricular ejection fraction by echocardiogram^b^ for high-risk CV patients**	✓	✓ (Consider if any signs or symptoms of HF, any atrial fibrillation/flutter)	
**Determination of baseline rhythm**	✓ (Baseline ECG)		
**Atrial fibrillation/flutter by pulse-check or ECG rhythm strip**	✓	✓ Transthoracic echocardiography if atrial fibrillation/flutter detected	✓ Consider a wearable device capable of monitoring rhythm

Incorporating recommendations from the 2022 European Society of Cardiology guidelines on cardio-oncology.^40^

a Strongly recommended to patients.

b A 3D-echocardiogram, which captures 3D views of the heart structures, is preferred 2D if available.

Abbreviations: 3D, 3-dimension; CV, cardiovascular; CVD, cardiovascular disease.

**Figure 1.**
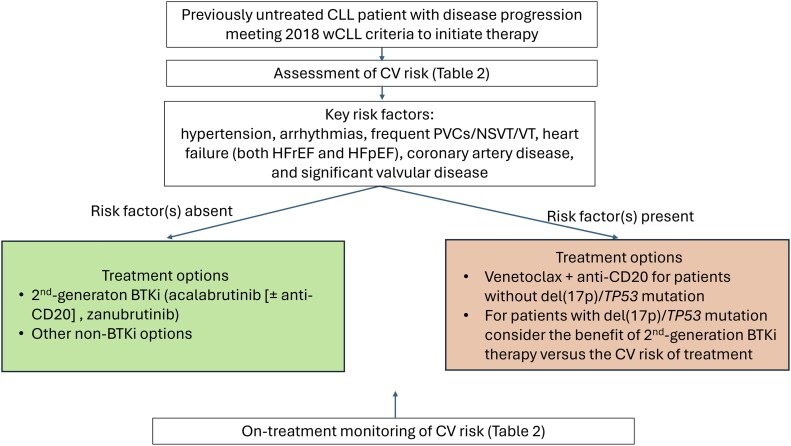
Management algorithm for patients with chronic lymphocytic leukemia by CV risk. Abbreviations: BTKi, Bruton tyrosine kinase inhibitor; CV, cardiovascular; HF, heart failure; HFpEF, heart failure with preserved ejection fraction; HFrEF, heart failure with reduced ejection fraction; iwCLL, International Workshop on Chronic Lymphocytic Leukemia; NSVT, non-sustained ventricular tachycardia; PVC, premature ventricular contractions; VT, ventricular tachycardia.

Pretreatment assessment can assist with placing patients into low- or high-risk category for the development of CV complications with a BTKi, and this determination can be used to inform treatment choice with the goal of balancing the risk of CV AEs and efficacy of cancer therapy. Key factors that identify patients at a high risk for CV AEs include having one or more of the following: hypertension, arrhythmias, frequent premature ventricular contractions/nonsustained ventricular tachycardia/ventricular tachycardia, heart failure (both heart failure with reduced ejection fraction and heart failure with preserved ejection fraction), coronary artery disease, and significant valvular disease (Figure 1). Acknowledging the limited comparative data among second-generation BTKis, first-line treatment recommendations are shown in Table 4.^8^^,^^10^^,^^41–43^

**Table 4.** Recommendations for first-line chronic lymphocytic leukemia treatment considering CV risk status and chronic lymphocytic leukemia genetic risk.

Patient CV risk and chronic ­lymphocytic leukemia genetic risk profile	Recommendation
CV low-risk Any chronic lymphocytic leukemia genetic risk	If a covalent BTKi is the preferred option, prescribe a second-generation BTKi (alone or in combination with an anti-CD20 for acalabrutinib) rather than first-generation ibrutinib to reduce the risk of CV AEs There are currently no data to suggest a difference in the CV AE profile between the second-generation BTKis, although data suggest a relatively high incidence of hypertension with zanubrutinib^8^^,^^10^ Also consider venetoclax in combination with an anti-CD20 monoclonal antibody, particularly for patients who prefer a time-limited therapy
CV high-risk Low-risk chronic lymphocytic leukemia genetic features (ie, no del(17p)/*TP53* mutation)	Consider a time-limited alternative to a second-generation BTKi, such as venetoclax in combination with obinutuzumab
CV high-risk High-risk chronic lymphocytic leukemia genetic features (ie, del(17p)/*TP53* mutation)	Based on efficacy data, a second-generation BTKi may be considered the more appropriate treatment, although decisions should be made on an individual basis after consultation with the patient. Anecdotal experience suggests BTKis should be used with caution for patients with a history of ventricular arrhythmias Data from the ELEVATE-TN (acalabrutinib ± obinutuzumab)^41^ and SEQUOIA (zanubrutinib ± venetoclax; non-randomized cohort)^42^ trials suggest the BTKi benefit is maintained in patients with high-risk genetic features Data from the CLL14 trial indicate that while patients with high-risk genetic features benefit from venetoclax + obinutuzumab, the benefit is not as durable as that achieved by those with low-risk genetic features^43^

Abbreviations: BTKi, Bruton tyrosine kinase inhibitor; CV, cardiovascular.

Potential drug–drug interactions are another important consideration, particularly because several frequently used antiarrhythmic agents and other CV medications may interact with BTKis. Beta-blockade is often preferred to CYP3A4 inhibitors (eg, verapamil and diltiazem) or P-glycoprotein substrates (amiodarone) to avoid interactions; a change in medication before starting BTKi therapy, or an alternative chronic lymphocytic leukemia therapy, should be considered for patients receiving such agents when alternative CV therapies are not feasible.^44^^,^^45^

### Management of bleeding risk with BTKis

Patients receiving anticoagulant or antiplatelet medication require specific consideration before the initiation of a BTKi, including review and potential alteration of their anticoagulation or antiplatelet regimen to an agent with a lower bleeding-risk profile. Limited data are available to inform this decision, primarily because patients at risk for bleeding are usually excluded from BTKi clinical trials, and there has been a tendency to consider anticoagulants and antiplatelets as a combined group when evaluating their relationship with BTKis and bleeding. Specifically, patients receiving warfarin were excluded from most clinical trials because of a fatal subdural hematoma in a patient taking warfarin in an early phase 1 trial with ibrutinib.^46^ The use of warfarin is generally considered a contraindication to BTKi treatment.

Available data suggest that clinically relevant doses of ibrutinib and acalabrutinib may potentiate the effects of antiplatelet therapies in some patients.^47^^,^^48^ Data from a single-center, retrospective study of patients receiving acalabrutinib for hematologic malignancies showed that taking an antiplatelet and/or anticoagulant significantly increased the risk of a clinically relevant major or minor bleed, while a nonsteroidal anti-inflammatory drug (NSAID) did not.^28^ In addition, current American College of Cardiology/American Heart Association/American College of Clinical Pharmacy/Heart Rhythm Society guidelines recommend direct oral anticoagulants, like apixaban, over warfarin for almost all patients with atrial fibrillation/flutter for the prevention of stroke or systemic embolism and a significantly lower risk of major bleeding.^49^

Suggestions for anticoagulant and antiplatelet agents to consider and those to avoid are shown in Table 5.^50^ Patients should also be cautioned against the use of fish oils, vitamin E, inadvertent use of aspirin‐containing products, and over-the-counter supplements such as turmeric. Finally, BTKis should be held before and after surgery to reduce the risk of periprocedural bleeding (Table 6).^30^

**Table 5.** Suggested cardiac based medications, anticoagulants, and antiplatelet based therapies to avoid/use with caution and those to consider for patients starting BTKi treatment.

Avoid or use with caution	Consider
Dual antiplatelet therapy (aspirin + clopidogrel/prasugrel/ticagrelor); if used, minimize the duration of therapy Warfarin (contraindicated) Non-dihydropyridine calcium-­channel blockers Dabigatran or edoxaban with pirtobrutinib due to p-glycoprotein inhibition (consider apixaban or rivaroxaban instead) Amiodarone/dronedarone Digoxin	Direct oral anticoagulants monotherapy for patients with stable ischemic heart disease or another indication for anticoagulation (atrial fibrillation/flutter),^50^ such as apixaban or rivaroxaban Beta blocker for hypertension or heart rate control

Abbreviation: BTKi, Bruton tyrosine kinase inhibitor.

**Table 6.** Management options for common BTKi-associated CV AEs.

CV toxicity	Management
**Atrial fibrillation**	Stroke-risk reduction Assess stroke risk using a validated risk score (CHADS_2_VaSc) and initiate anticoagulation with a direct oral anticoagulants per standard practice Consider additional risk factors for stroke not included in CHADS_2_VaSc (left atrial dilatation, uncontrolled hypertension, proteinuria, chronic kidney disease, prior venous thromboembolism/deep-vein thrombosis, obesity, other malignancies) when considering the risk/benefits of anticoagulation Rate vs rhythm control For rate control, beta blockers are preferred to calcium channel blockers (because of the potential drug–drug interactions with the non-dihydropyridine calcium channel blockers) For rhythm control, consider potential drug–drug interactions with antiarrhythmic drugs; also consider a rhythm control strategy with electrical cardioversion and/or catheter ablation (particularly for symptomatic patients) Consider/discuss early rhythm control if the patient is age <60 years to reduce atrial fibrillation/flutter burden/remodeling (atrial myopathy) Consider rhythm control for patients with heart failure Other considerations An echocardiogram for all patients with new onset/newly diagnosed atrial fibrillation/flutter to assess valves, atrial size, and left ventricular function Monitor rhythm to assess the burden of atrial fibrillation/flutter, which can inform decision-making and help to refine risk Identify opportunities to reduce bleeding risk (control blood pressure, eliminate aspirin, P2Y12 receptor blockers, NSAIDs, and supplements [fish oil, etc.]) Shared decision-making with the patient considering the “net clinical benefit” of anticoagulation for stroke prevention vs. bleeding risk
**Hypertension**	For patients with low potassium and normal renal function, or history of diabetes or heart failure with reduced ejection fraction, consider an ACE inhibitor or ARB Consider a beta blocker that increases nitric oxide, such as carvedilol and nebivolol, because of the potential for down regulation of nitric oxide formation Consider a dihydropyridine calcium-channel blocker, such as amlodipine or nifedipine Assess for proteinuria in patients with new-onset or newly uncontrolled hypertension; for patients with proteinuria, consider an ACE inhibitor or ARB Pay careful attention to drug–drug interactions
**Bleeding**	Carefully consider potential drug–drug interactions Hold BTKi for 3 days (minor procedure) or 7 days (major procedure) before and after surgery to reduce the risk of periprocedural bleeding^30^ For all patients: Assess for potential precipitants/reversible causes (gastrointestinal source, trauma, etc.) Temporarily hold medication until bleeding stops For patients on anticoagulation (for atrial fibrillation): Hold anticoagulant until bleeding stops If the patient has spontaneous bleeding/severe bruising, consider a reduced-intensity direct oral anticoagulant (apixaban 2.5 mg BID or rivaroxaban 10 mg daily), although there is currently no evidence to support this approach If the patient is unable to tolerate consistent anticoagulation and has a high stroke risk, consider a left atrial appendage occluder device If stroke risk is low (CHADS_2_VaSc 1-2 [2-3 for women]), consider withholding anticoagulation after discussion/shared decision-making with the patient Consider monitoring rhythm with a topical monitor to assess “burden of atrial fibrillation” to refine risk and inform decision-making

Abbreviations: ACE, angiotensin-converting enzyme; AE, adverse event; AF, atrial fibrillation/flutter; ARB, angiotensin II receptor blocker; BID, twice daily; BTKi, Bruton tyrosine kinase inhibitor; CV, cardiovascular; HFrEF, heart failure with reduced ejection fraction; P2Y12, P2Y purinoceptor 12.

## Considerations for managing patients with new-onset or worsening CV events during chronic lymphocytic leukemia treatment

For patients doing well on chronic lymphocytic leukemia therapy, the goal is to keep them on treatment. For some patients, it may be necessary to temporarily discontinue the BTKi and manage the CV event. When the clinical event is resolved or controlled, a patient can typically resume BTKi treatment if needed. For patients with an event that is not adequately controlled, continuation of the particular BTKi, even with dose reduction, is not recommended, and another BTKi or an alternative class of therapy should be considered (such as venetoclax plus an anti-CD20 monoclonal antibody). With respect to managing the CV event, best practice is to have a multidisciplinary discussion and apply the key principles and options summarized in Table 6 and in other publications.^44^^,^^45^^,^^51^

To ensure the best outcome, a collaborative care approach is essential, including hematologists, cardiologists/cardio-oncologists, nurses, pharmacists, and often primary care providers. It may be necessary to refer patients at high risk for a CV event or who develop a CV event to a cardiologist or cardio-oncologist for specialized management. For most patients, ongoing management by their own cardiologist in collaboration with the hematology team is an appropriate approach. Triggers for referring a patient to a cardiologist or cardio-oncologist include the development of any of the following: a new arrhythmia (symptomatic or asymptomatic), left ventricular dysfunction (symptomatic or asymptomatic), difficult to control hypertension, or any new symptoms concerning cardiac etiology.

## Conclusions

Baseline and ongoing CV risk assessment, careful monitoring, management (including attention to potential drug–drug interactions), and a multidisciplinary team approach are all critical to ensure optimal oncologic and CV outcomes for patients with chronic lymphocytic leukemia, and potentially other hematological malignancies, receiving BTKis. Hypertension and bleeding risk are particularly important considerations for patients with chronic lymphocytic leukemia who are often older and may exhibit intrinsic bruising and/or bleeding tendencies because of disease-specific effects. For patients with a high CV risk, or those developing CV AEs while taking a BTKi, selecting treatment for chronic lymphocytic leukemia with the least CV risk is important. Second-generation BTKis are safer than ibrutinib, especially with respect to arrhythmias. The role of noncovalent BTKis, which may be associated with fewer CV AEs, remains to be fully established, but holds promise.

There are many areas in this clinical space that remain uncertain and will require improvement or investigation to optimize patient outcomes. Improving trial designs to consistently and robustly assess CV risk at baseline will provide data to better inform the management of at-risk patients. The inclusion of detailed information relating to the detection and classification of minor and major bleeding will increase understanding about these AEs. Gaps that require investigation include the impact of BTKi dose/duration on CV risk; the risk of embolic stroke with BTKis and the effect of anticoagulants and antiplatelets; the relationship between the chronic lymphocytic leukemia genetic risk factors like immunoglobulin heavy chain variable gene mutation and chromosomal abnormalities and the risk of CV AEs; and potential preventive strategies to mitigate CV AEs.^52^

## Data Availability

Not applicable.

## References

[1] Mato AR , Woyach JA, Brown JR, et al Pirtobrutinib after a covalent BTK inhibitor in chronic lymphocytic leukemia. N Engl J Med. 2023;389:33-44. 10.1056/NEJMoa2300696 37407001

[2] Woyach JA , Stephens DM, Flinn IW, et al First-in-human study of the reversible BTK inhibitor nemtabrutinib in patients with relapsed/refractory chronic lymphocytic leukemia and B-cell non-Hodgkin lymphoma. Cancer Discov. 2024;14:66-75. 10.1158/2159-8290.CD-23-0670 37930156

[3] Coombs CC. Frontline therapy of CLL-changing treatment paradigms. Curr Hematol Malig Rep. 2024;19:65-74. 10.1007/s11899-024-00726-x 38337108

[4] Bennett R , Seymour JF. Update on the management of relapsed/refractory chronic lymphocytic leukemia. Blood Cancer J. 2024;14:33. 10.1038/s41408-024-01001-1 38378673 PMC10879527

[5] Referenced with permission from the NCCN Clinical Practice Guidelines in Oncology (NCCN Guidelines^®^) for Chronic Lymphocytic Leukemia/Small Lymphocytic Lymphoma V.2.2025. © National Comprehensive Cancer Network, Inc. 2024. All rights reserved. Accessed March 3, 2025. To view the most recent and complete version of the guideline, go online to NCCN.org.

[6] Garg N , Padron EJ, Rammohan KW, et al Bruton’s tyrosine kinase inhibitors: the next frontier of B-cell-targeted therapies for cancer, autoimmune disorders, and multiple sclerosis. J Clin Med. 2022;11:6139. 10.3390/jcm11206139 36294458 PMC9604914

[7] Tam C , Thompson PA. BTK inhibitors in CLL: second-generation drugs and beyond. Blood Adv. 2024;8:2300-2309. 38478390 10.1182/bloodadvances.2023012221 PMC11117011

[8] Seymour JF , Byrd JC, Ghia P, et al Detailed safety profile of acalabrutinib vs ibrutinib in previously treated chronic lymphocytic leukemia in the ELEVATE-RR trial. Blood. 2023;142:687-699. 10.1182/blood.2022018818 37390310 PMC10644206

[9] Byrd JC , Hillmen P, Ghia P, et al Acalabrutinib versus ibrutinib in previously treated chronic lymphocytic leukemia: results of the first randomized phase III trial. J Clin Oncol. 2021;39:3441-3452. 10.1200/JCO.21.01210 34310172 PMC8547923

[10] Brown JR , Eichhorst B, Hillmen P, et al Zanubrutinib or ibrutinib in relapsed or refractory chronic lymphocytic leukemia. N Engl J Med. 2023;388:319-332. 10.1056/NEJMoa2211582 36511784

[11] Brown JR , Byrd JC, Ghia P, et al Cardiovascular adverse events in patients with chronic lymphocytic leukemia receiving acalabrutinib monotherapy: pooled analysis of 762 patients. Haematologica. 2022;107:1335-1346. 10.3324/haematol.2021.278901 34587719 PMC9152976

[12] Chen ST , Azali L, Rosen L, et al Hypertension and incident cardiovascular events after next-generation BTKi therapy initiation. J Hematol Oncol. 2022;15:92. 10.1186/s13045-022-01302-7 35836241 PMC9281099

[13] Common Terminology Criteria for Adverse Events (CTCAE) v5.0. Accessed July 2024. https://ctep.cancer.gov/protocolDevelopment/electronic_applications/ctc.htm

[14] Diamond A , Bensken WP, Vu L, Dong W, Koroukian SM, Caimi P. Ibrutinib is associated with increased cardiovascular events and major bleeding in older CLL patients. JACC CardioOncol. 2023;5:233-243. 10.1016/j.jaccao.2023.02.001 37144107 PMC10152196

[15] Mato A , Tang B, Azmi S, et al A real-world study to assess the association of cardiovascular adverse events (CVAEs) with ibrutinib as first-line (1L) treatment for patients with chronic lymphocytic leukaemia (CLL) in the United States. E J Haem. 2023;4:135-144. 10.1002/jha2.638 PMC9928661 36819172

[16] Htut TW , Han MM, Thein KZ. Acalabrutinib-related cardiac toxicities in patients with chronic lymphocytic leukemia: a meta-analysis of randomized controlled trials. J Immunother Precis Oncol. 2022;5:43-47. 10.36401/JIPO-21-12 35664088 PMC9153250

[17] Dimopoulos MA , Opat S, D’Sa S, et al Zanubrutinib versus ibrutinib in symptomatic Waldenström macroglobulinemia: final analysis from the randomized phase III ASPEN study. J Clin Oncol. 2023;41:5099-5106. 10.1200/JCO.22.02830 37478390 PMC10666987

[18] Moslehi JJ , Furman RR, Tam CS, et al Cardiovascular events reported in patients with B-cell malignancies treated with zanubrutinib. Blood Adv. 2024;8:2478-2490. 10.1182/bloodadvances.2023011641 38502198 PMC11131064

[19] Lampson BL , Yu L, Glynn RJ, et al Ventricular arrhythmias and sudden death in patients taking ibrutinib. Blood. 2017;129:2581-2584. 10.1182/blood-2016-10-742437 28223277 PMC7219062

[20] Guha A , Derbala MH, Zhao Q, et al Ventricular arrhythmias following ibrutinib initiation for lymphoid malignancies. J Am Coll Cardiol. 2018;72:697-698. 10.1016/j.jacc.2018.06.002 30072003 PMC7529121

[21] Bhat SA , Gambril J, Azali L, et al Ventricular arrhythmias and sudden death events following acalabrutinib initiation. Blood. 2022;140:2142-2145. 10.1182/blood.2022016953 35917449 PMC10405526

[22] Kater AP , Owen C, Moreno C, et al Fixed-duration ibrutinib-venetoclax in patients with chronic lymphocytic leukemia and comorbidities. NEJM Evid. 2022;1:EVIDoa2200006. 10.1056/EVIDoa2200006 38319255

[23] Woyach JA , Ruppert AS, Heerema NA, et al Ibrutinib regimens versus chemoimmunotherapy in older patients with untreated CLL. N Engl J Med. 2018;379:2517-2528. 10.1056/NEJMoa1812836 30501481 PMC6325637

[24] Sharman JP , Ghia P, Miranda P, et al Analysis of ventricular arrhythmias and sudden death from prospective, randomized clinical trials of acalabrutinib. Br J Haematol. 2024;205:529-533. 10.1111/bjh.19469 38634256

[25] Lip GYH , Coca A, Kahan T, et al Hypertension and cardiac arrhythmias: a consensus document from the European Heart Rhythm Association (EHRA) and ESC Council on Hypertension, endorsed by the Heart Rhythm Society (HRS), Asia-Pacific Heart Rhythm Society (APHRS) and Sociedad Latinoamericana de Estimulación Cardíaca y Electrofisiología (SOLEACE). Europace. 2017;19:891-911. 10.1093/europace/eux091 28881872

[26] Rao MP , Halvorsen S, Wojdyla D, et al Apixaban for Reduction in Stroke and Other Thromboembolic Events in Atrial Fibrillation (ARISTOTLE) Steering Committee and Investigators. Blood pressure control and risk of stroke or systemic embolism in patients with atrial fibrillation: results from the apixaban for reduction in stroke and other thromboembolic events in atrial fibrillation (ARISTOTLE) trial. J Am Heart Assoc. 2015;4: e002015. 10.1161/JAHA.115.002015 26627878 PMC4845276

[27] Caldeira D , Alves D, Costa J, Ferreira JJ, Pinto FJ. Ibrutinib increases the risk of hypertension and atrial fibrillation: systematic review and meta-analysis. PLoS One. 2019;14:e0211228. 10.1371/journal.pone.0211228 30785921 PMC6382095

[28] Kumar PS , Wiczer T, Rosen L, et al Evaluation of bleeding events in patients receiving acalabrutinib therapy. Leukemia. 2023;37:1554-1557. 10.1038/s41375-023-01869-1 36932166

[29] Wang T-F , Baumann Kreuziger L, Leader A, et al Characteristics and outcomes of patients on concurrent direct oral anticoagulants and targeted anticancer therapies-TacDOAC registry: communication from the ISTH SSC subcommittee on hemostasis and malignancy. J Thromb Haemost. 2021;19:2068-2081. 10.1111/jth.15367 34327825

[30] Lipsky A , Lamanna N. Managing toxicities of Bruton tyrosine kinase inhibitors. Hematology Am Soc Hematol Educ Program. 2020;2020:336-345. 10.1182/hematology.2020000118 33275698 PMC7727553

[31] Busygina K , Jamasbi J, Seiler T, et al Oral Bruton tyrosine kinase inhibitors selectively block atherosclerotic plaque-triggered thrombus formation in humans. Blood. 2018;131:2605-2616. 10.1182/blood-2017-09-808808 29559479

[32] National Library of Medicine. A study of pirtobrutinib (LOXO-305) versus ibrutinib in participants with chronic lymphocytic leukemia (CLL)/small lymphocytic lymphoma (SLL) (BRUIN-CLL-314). Accessed June 20, 2024. https://www.clinicaltrials.gov/study/NCT05254743

[33] Staerk L , Wang B, Preis SR, et al Lifetime risk of atrial fibrillation according to optimal, borderline, or elevated levels of risk factors: cohort study based on longitudinal data from the Framingham Heart Study. BMJ. 2018:361: k1453. 10.1136/bmj.k1453 29699974 PMC5917175

[34] Kornej J , Börschel CS, Benjamin EJ, Schnabel RB. Epidemiology of atrial fibrillation in the 21st century: novel methods and new insights. Circ Res. 2020;127:4-20. 10.1161/CIRCRESAHA.120.316340 32716709 PMC7577553

[35] Shanafelt TD , Parikh SA, Noseworthy PA, et al Atrial fibrillation in patients with chronic lymphocytic leukemia (CLL). Leuk Lymphoma. 2017;58:1630-1639. 10.1080/10428194.2016.1257795 27885886

[36] Avalon JC , Fuqua J, Miller T, et al Pre-existing cardiovascular disease increases risk of atrial arrhythmia and mortality in cancer patients treated with ibrutinib. Cardiooncology. 2021;7:38. 10.1186/s40959-021-00125-8 34798905 PMC8603583

[37] Fernandez Turizo MJ , Kim E, Asnani A, Yankama T, von Keudell G, Mejías-De Jesús C. Pre-existing cardiovascular disease increases the risk of cardiovascular adverse events during Bruton tyrosine kinase inhibitor therapy. Blood. 2023;142:496. 10.1182/blood-2023-174560 PMC11886567 39244718

[38] Azali L , Hazelden L, Wiczer T, et al Evaluation of the incidence and risk factors associated with major cardiovascular events in patients receiving acalabrutinib therapy. Blood. 2020;136:29-30.

[39] Dickerson T , Wiczer T, Waller A, et al Hypertension and incident cardiovascular events following ibrutinib initiation. Blood. 2019;134:1919-1928. 10.1182/blood.2019000840 31582362 PMC6887116

[40] Lyon AR , López-Fernández T, Couch LS, et al ESC Scientific Document Group. 2022 ESC guidelines on cardio-oncology developed in collaboration with the European Hematology Association (EHA), the European Society for Therapeutic Radiology and Oncology (ESTRO) and the International Cardio-Oncology Society ­(IC-OS). Eur Heart J. 2022;43:4229-4361. 10.1093/eurheartj/ehac244 36017568

[41] Sharman JP , Egyed M, Jurczak W, et al Acalabrutinib ± obinutuzumab vs obinutuzumab + chlorambucil in treatment-naive chronic lymphocytic leukemia: 6-year follow-up of elevate-TN. Blood. 2023;142:636. 10.1182/blood-2023-174750

[42] Tam CS , Brown JR, Kahl BS, et al Zanubrutinib versus bendamustine and rituximab in untreated chronic lymphocytic leukaemia and small lymphocytic lymphoma (SEQUOIA): a randomised, controlled, phase 3 trial. Lancet Oncol. 2022;23:1031-1043. 10.1016/S1470-2045(22)00293-5 35810754

[43] Al-Sawaf O , Robrecht S, Zhang C, et al Venetoclax-obinutuzumab for previously untreated chronic lymphocytic leukemia: 6-year results of the randomized CLL14 study. Hemasphere. 2023;7:e064430a. 10.1097/01.HS9.0000967492.06443.0a PMC11551846 39082668

[44] Quartermaine C , Ghazi SM, Yasin A, et al Cardiovascular toxicities of BTK inhibitors in chronic lymphocytic leukemia. JACC CardioOncol. 2023;5:570-590. 10.1016/j.jaccao.2023.09.002 37969643 PMC10635896

[45] Aghel N , Baro Vila RC, Lui M, Hillis C, Leong DP. Diagnosis and management of cardiovascular effects of Bruton’s tyrosine kinase inhibitors. Curr Cardiol Rep. 2023;25:941-958. 10.1007/s11886-023-01916-4 37498449

[46] Byrd JC , Brown JR, O’Brien S, et al RESONATE Investigators. Ibrutinib versus ofatumumab in previously treated chronic lymphoid leukemia. N Engl J Med. 2014;371:213-223. 10.1056/NEJMoa1400376 24881631 PMC4134521

[47] Series J , Garcia C, Levade M, et al Differences and similarities in the effects of ibrutinib and acalabrutinib on platelet functions. Haematologica. 2019;104:2292-2299. 10.3324/haematol.2018.207183 30819914 PMC6821604

[48] Bye AP , Unsworth AJ, Vaiyapuri S, Stainer AR, Fry MJ, Gibbins JM. Ibrutinib inhibits platelet integrin αIIbβ3 outside-in signaling and thrombus stability but not adhesion to collagen. Arterioscler Thromb Vasc Biol. 2015;35:2326-2335. 10.1161/ATVBAHA.115.306130 26359510

[49] Joglar JA , Chung MK, Armbruster AL, et al Peer Review Committee Members. 2023 ACC/AHA/ACCP/HRS guideline for the diagnosis and management of atrial fibrillation: a report of the American College of Cardiology/American Heart Association Joint Committee on clinical practice guidelines. Circulation. 2024;149:e1-e156. 10.1161/CIR.0000000000001193 38033089 PMC11095842

[50] Yasuda S , Kaikita K, Akao M, et al AFIRE Investigators. Antithrombotic therapy for atrial fibrillation with stable coronary disease. N Engl J Med. 2019;381:1103-1113. 10.1056/NEJMoa1904143 31475793

[51] Awan FT , Addison D, Alfraih F, et al International consensus statement on the management of cardiovascular risk of Bruton’s tyrosine kinase inhibitors in CLL. Blood Adv. 2022;6:5516-5525. 10.1182/bloodadvances.2022007938 35790105 PMC9631706

[52] Dent S , Rader RK, White O, Patterson B, Moore HN. Moving beyond cardiotoxicity detection to prevention: a pharmacologic review. Curr Treat Options Cardio Med. 2024;26:1-12. 10.1007/s11936-023-01030-2

